# Supplementary material for: Evaluating the safety of prenatal HIV PrEP use: Perinatal outcomes from three cohort studies in Western Kenya
Source: PLOS Glob Public Health. 2026 Jan 20;6(1):e0005744. doi: 10.1371/journal.pgph.0005744 (PMC12818637; doi:10.1371/journal.pgph.0005744)
Supplement: Attachment — Submitted filename: PLoS GPH_Odhiambo_PrEP safety_Response to editor.docx [file pgph.0005744.s004.docx]

December 11, 2025

Everton Falcão de Oliveira

Academic Editor
*PLOS Global Public Health*

Dear Dr. Oliveira,

Thank you for your careful review of our original research manuscript, “*Evaluating the Safety of Prenatal HIV PrEP Use: Perinatal Outcomes from Three Cohort Studies in Western Kenya”* (Submission ID: PGPH-D-25-02190). Based on your suggestions we have updated the following parts of the submission:

1. We have updated the financial disclosure section to reflect the grant, grand number and who it was awarded to.
2. We have included a List of Legends which includes figure, table, and supplement file names
3. We have removed identifying information, such as exact dates, from our de-identified dataset.

The clean version of the manuscript and updated supplemental files have been re-uploaded. Thank you for your review and consideration of our revised manuscript.

Sincerely,

Jillian Pintye, RN, MPH, PhD
Associate Professor

Department of Biobehavioral Nursing & Health Informatics | School of Nursing

Department of Global Health | School of Public Health
